# The reliability and validity of PHQ-9 in patients with major depressive disorder in psychiatric hospital

**DOI:** 10.1186/s12888-020-02885-6

**Published:** 2020-09-29

**Authors:** Yue Sun, Zhaoyan Fu, Qijing Bo, Zhen Mao, Xin Ma, Chuanyue Wang

**Affiliations:** 1 The National Clinical Research Center for Mental Disorders & Beijing Key Laboratory of Mental Disorders & Beijing Institute for Brain Disorders Center of Schizophrenia, Beijing Anding Hospital, Capital Medical University, No.5 Ankang Lane, Dewai Avenue, Xicheng District, Beijing, 100088 China; 2 Advanced Innovation Center for Human Brain Protection, Capital Medical University, Beijing, 100069 China

**Keywords:** PHQ-9, Reliability, Validity, Major depressive disorder

## Abstract

**Background:**

To assess the reliability and validity of Patient Health Questionnaire-9 (PHQ-9) for patients with major depressive disorder (MDD) and to assess the feasibility of its use in psychiatric hospitals in China.

**Methods:**

One hundred nine outpatients or inpatients with MDD who qualified the Diagnostic and Statistical Manual of Mental Disorders, fourth edition (DSM-IV) criteria completed PHQ-9 and Hamilton Depression Scale (HAMD-17). Two weeks after the initial evaluation, 54 randomly selected patients underwent repeat assessment using PHQ-9. For validity analysis, the construct validity and criterion validity were assessed. The internal concordance coefficient and the test-retest correlation coefficients were used for reliability analysis. The correlation between total score and scores for each item and the correlation between scores for various items were evaluated using Pearson correlation coefficient.

**Results:**

Principal components factor analysis showed good construct validity of the PHQ-9. PHQ-9 total score showed a positive correlation with HAMD-17 total score (*r* = 0.610, *P* < 0.001). With HAMD as the standard, PHQ-9 depression scores of 7, 15, and 21 points were used as cut-offs for mild, moderate, and severe depression, respectively. Consistency assessment was conducted between the depression severity as assessed by PHQ-9 and HAMD (Kappa = 0.229, *P* < 0.001). Intraclass correlation coefficient between PHQ-9 total score and HAMD total score was 0.594 (95% confidence interval, 0.456–0.704, *P* < 0.001). The Cronbach’s α coefficient of PHQ-9 was 0.892. Correlation coefficients between each item score and the total score ranged from 0.567–0.789 (*P* < 0.01); the correlation coefficient between various item scores ranged from 0.233–0.747. The test-retest correlation coefficient for total score was 0.737.

**Conclusions:**

PHQ-9 showed good reliability and validity, and high adaptability for patients with MDD in psychiatric hospital. It is a simple, rapid, effective, and reliable tool for screening and evaluation of the severity of depression.

## Background

Major depressive disorder (MDD) is a common chronic recurrent mental disease. According to the Global Burden of Disease Study (2015), MDD accounted for 35% of disability-adjusted life years (DALYs) and ranked first among the psychiatric disorders [1]. Comprehensive and systematic treatment of MDD is a key imperative throughout the disease course. Measurement-based care is an emerging paradigm of care for patients with MDD; moreover, it can facilitate early detection of depression, help monitor the changes in clinical symptoms, and guide treatment decision-making. The guidelines of the American Psychological Association for MDD treatment emphasize the importance of accurate disease evaluation and monitoring of therapeutic response throughout the treatment [2–4]. Therefore, identification of a convenient and effective screening tool to monitor the treatment effect and the severity of depression may help improve the management of MDD.

PHQ-9 is a rapid and effective tool for detection as well as for monitoring the severity of depression [5]. It has been widely used in community-based settings, in the general population, and among people with physical diseases [6–10]. In a meta-analysis, the reliability and validity of PHQ-9 was found to be better than that of DSM-IV (Diagnostic and Statistical Manual of Mental Disorders, fourth edition) [11, 12]. A study of 6000 subjects found that PHQ-9 is more than a screening tool for depression; it is also a reliable and effective tool for monitoring the severity of depression [13]. Various versions of PHQ-9 have been developed in different languages, including Chinese, French, Spanish, Arabic, Korean, Somali, Thai, and Greek [14–20]. A meta-analysis of 17 studies concluded that PHQ-9 is suitable for use in different populations in different countries [11]. The reliability and validity of PHQ-9 (Chinese version) as a screening tool for depression has been validated in large studies conducted in Hong Kong (*n* = 6028) and Taiwan (*n* = 1954) [21, 22]. The effectiveness of PHQ-9 is supported by other related studies conducted in China [23–28].

Most of the domestic and overseas studies pertaining to PHQ-9 were conducted in community-based primary medical care institutions or in the general population; however, few studies have been conducted in psychiatric hospitals. In psychiatric hospitals, patients with depression have more severe disease and manifest complex symptoms. Self-assessment questionnaire for depression can help improve the detection of depression, especially in patients who have other mental disorders with comorbid depression episodes. In a study of 153 outpatients at a Japanese psychiatric hospital, PHQ-9 was found to be helpful for screening, but not suitable for diagnosing depressive episode [29].

The purpose of this study was to verify the feasibility of use of PHQ-9 in psychiatric hospitals in China and to test the reliability and validity of its use in patients with MDD.

## Methods

### Subjects

A total of 109 patients with MDD (including both outpatients and inpatients) were recruited at the Beijing Anding hospital, Capital Medical University. The inclusion criteria were: 1) patients who qualified the DSM-IV criteria for MDD [patients were diagnosed using the Structured Clinical Interview for DSM (SCID)]; 2) male or female patients aged 16–55 years; 3) patients with secondary education or above (at least 9 years of education); 4) no history of electroconvulsive therapy (MECT) during the last 3 months; 5) provision of written informed consent for participation by patients and/or guardians after detailed counseling.

The exclusion criteria were: 1) patients with organic brain disease or severe, unstable physical disease which significantly affects the treatment of mental disorder; 2) patients with secondary depression (physical disease, drug-induced, or other mental disease); 3) patients with serious drug side effects that required urgent redressal; 4) patients with serious suicide attempt; 5) pregnant women.

### Instruments

#### Patient health questionnaire-9

The PHQ-9 was used as a self-administered, screening tool for assessment of the severity of depressive symptoms. Unlike other depression scales, PHQ-9 includes 9 items which focus on the Diagnostic and Statistical Manual of Mental Disorders, 4th edition (DSM-IV) for MDD. The questionnaire assesses how often the subjects had been disturbed by any of the 9 items during the immediately preceding 2 weeks.

Each item of PHQ-9 was scored on a scale of 0–3 (0 = not at all; 1 = several days; 2 = more than a week; 3 = nearly every day). The PHQ-9 total score ranges from 0 to 27 (scores of 5–9 are classified as mild depression; 10–14 as moderate depression; 15–19 as moderately severe depression; ≥ 20 as severe depression) [30].

#### Hamilton depression scale-17

HAMD-17 is a widely used tool for assessment of the severity of depression. The scale contains 17 items, each of which is scored on a scale of 0–4 (0 represents asymptomatic and 1–4 represent symptomatic). Total scores of HAMD-17 range from 0 to 52: scores of 0–7 are defined as normal; 8–16 are considered as mild depression; 17–23 as moderate depression; and > 24 as severe depression [31].

### Procedures

**Fig. 1:**
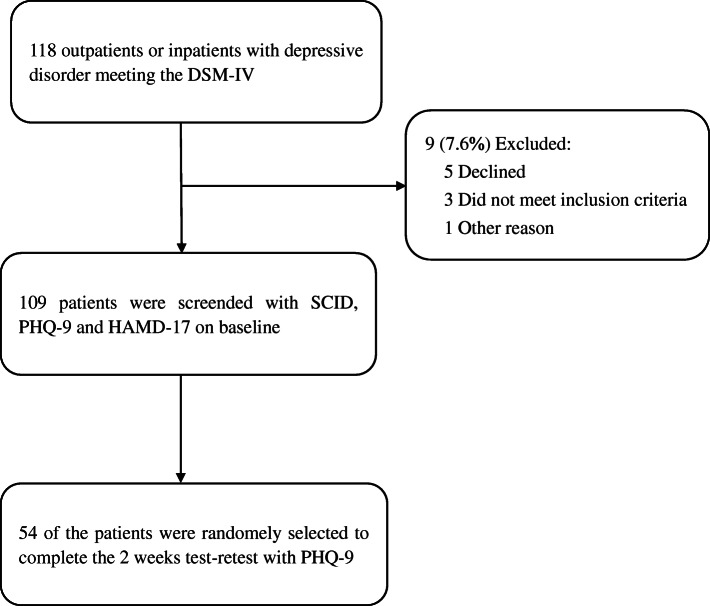
Schematic illustration of the study design and patient-selection criteria. PHQ-9: Patient Health Questionnaire 9 items, Hamilton Rating Scale for Depression; PCC, primary care clinic; HAMD-17: Hamilton Depression Scale 17 items; DSM-IV: Diagnostic and Statistical Manual of Mental Disorders, fourth edition

One hundred eighteen outpatients or inpatients with MDD qualified the DSM-IV criteria. Of these, 9 patients were excluded: 5 subjects declined to participate in this study, 3 subjects did not qualify the inclusion criteria, and 1 subject had other reason. Finally, 109 patients with major depressive disorder completed the PHQ-9 and Hamilton Depression Scale (HAMD-17). Of these, 54 patients were randomly selected to undergo a repeat test with PHQ-9, 2 weeks after the initial assessment (Fig. 1).

### Statistical analysis

Data entry and processing were performed using Epi-data 3.1. Data analysis was performed using Statistical Product and Service Solutions version 23.0 (SPSS 23.0). Between-group differences with respect to continuous variables were assessed using the *t* test; those with respect to dichotomous variables were assessed using the Chi-squared test. Cronbach’s α coefficient and Pearson’s correlation coefficient was used to analyze the internal concordance coefficient and the test-retest correlation coefficient, respectively, for reliability analysis. The correlation among each item score and the correlation of each item score with the total score were evaluated using the Pearson correlation coefficient. Intraclass correlation coefficient (ICC) and Kappa analysis were used for consistency test. ICC is equal to the individual variance divided by the total variance; therefore, its value ranges from 0 to 1: 0 represents poor trust; 1 represents perfect trust. It is generally believed that a reliability coefficient < 0.4 represents poor reliability, while > 0.75 represents good reliability [32]. For the validity analysis, the criterion validity and construct validity of PHQ-9 were assessed with factor analysis and correlation analysis, respectively.

## Results

### Demographic and clinical characteristics of the study population

The mean age of 109 patients was 34.86 ± 10.90 years (range, 16–55); these included 54 males and 55 females. The number of years of education ranged from 9 to 22 years (mean: 13.10 ± 3.09 years). The total course of disease ranged from 1 to 396 months (mean disease course: 67.78 ± 70.79 months) (Table 1).

**Table 1:** Demographic and clinical characteristics of the study population

Characteristics	Baseline (*n* = 109)	Repeat assessment at 2 weeks (*n* = 54)	PHQ-9 Score M (SD)		HAMD-17 Score M (SD)
			Baseline	2-week	Baseline
	N (%)	N (%)			
Gender					
Male	54 (49.5)	23 (42.6)	11.87 (8.4)	12.22 (7.78)	14.87 (16.05)
Female	55 (50.5)	31 (57.4)	9.61 (7.09)	9.03 (7.21)	9.06 (9.18)
Marital status					
Never married	55 (50.5)	28 (51.9)	8.39 (7.42)	8.32 (6.95)	11.00 (12.71)
Married/Cohabit	48 (44.0)	23 (42.6)	12.26 (7.07)	12.87 (7.74)	12.98 (14.44)
Separated/Divorced	5 (4.6)	2 (3.7)	16.50 (13.44)	9.00 (11.31)	9.40 (8.36)
Widowed	1 (0.9)	1 (1.9)	21.0	14.00	28
	Mean (SD)	Mean (SD)			
Age (years)	34.86 (10.90)	34.26 (10.94)			
Duration of illness (months)	67.78 (70.79)	61.17 (59.46)			
Education (years)	13.10 (3.0)	13.35 (3.18)			

*PHQ-9* Patient Health Questionnaire, *HAMD* Hamilton Depression Scale, *SD* Standard deviation

### Validity

On assessment of the consistency between total HAMD scores and total PHQ-9 scores, the intraclass correlation coefficient was 0.594 (> 0.4, moderate) [95% confidence interval (CI): 0.456–0.704, *P* < 0.001]. The cut-off points of PHQ-9 depression scores for mild, moderate, and severe disease were 7, 15, and 21 points, respectively. Based on the cut-off points, consistency analysis between the depression severity obtained by PHQ-9 and HAMD revealed a Kappa score of 0.229 (*P* < 0.001). The correlation between severity scores of the two scales was general and statistically significant. On internal consistency test, the standard Cronbach’s α coefficient for PHQ-9 was 0.892.

The KOM and Bartlett’s test of sphericity showed that all items in the PHQ-9 were correlated with each other, and the data structure was reasonable [KOM test coefficient: 0.895; KOM test coefficients of individual variables were > 0.8 (range of KOM test coefficient: 0.859–0.930); Bartlett’s test result was *P* < 0.001]; this indicated that the data was suitable for principal component analysis. On principal component analysis, the eigenvalues of the first two principal components were > 1, which explained the total data variation of 54.505 and 11.406%, respectively. However, based on the scree plot test and interpretion of the results, the principal components factor analysis, method of varimax, supported one factor structure; the eigenvalue was 4.91 and the percent variance was 54.51%, which indicated that all the items in PHQ-9 exhibited the same problem. All factor load matrix coefficients of each item were > 0.5 (range of loadings: 0.55–0.85). The total scores of the HAMD showed a positive correlation with the total scores of the PHQ-9 (*r* = 0.610, *P* < 0.001).

### Cut-off points of PHQ-9 for depression severity

**Fig. 2:**
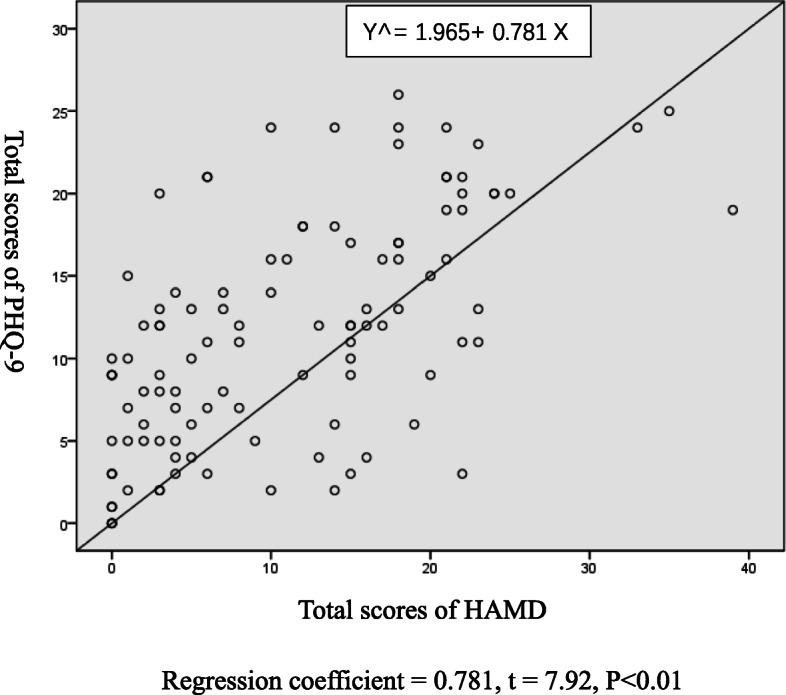
Regression line graph of PHQ-9 total scores and HAMD total scores. PHQ-9: Patient Health Questionnaire; HAMD: Hamilton Depression Scale

Using total score of HAMD as the independent variable, linear regression analysis of total score of HAMD and total score of PHQ-9 was performed (Fig. 2). Using the total score of HAMD as independent variable X and the total score of PHQ-9 as the dependent variable Y, the regression equation was Y^= 1.965+ 0.781 X. *t* test was conducted on regression coefficient 0.781, *t* = 7.92 (*P* < 0.01), and regression relation was observed between the total HAMD score and total PHQ-9 score. The coefficient of determination *R*^2^ = 0.374 and the regression model showed a good fit. Cut-off points of 7, 17, and 24 on HAMD scale represented mild, moderate, and severe symptom levels; the corresponding cut-off points on PHQ-9 scale were 7, 15, and 21, respectively.

### Reliability

Two weeks after the initial assessment, 54 patients underwent repeat assessment using PHQ-9. Pearson correlation analysis showed a test-retest reliability coefficient of 0.737 for the total scores (*P* < 0.01); the test-retest reliability coefficient for each item score ranged from 0.552–0.728 (*P* < 0.01). These findings indicated a significant correlation between the scores of the two tests.

### Correlation analysis between each item and total scale score of PHQ-9

Pearson correlation analysis was used to assess the correlation of each item score of PHQ-9 with the total score; the correlation coefficients ranged from 0.567–0.789 (*P* < 0.01). The correlation coefficients of each item ranged from 0.233–0.747 (Table 2).

**Table 2:** Correlation coefficients showing the correlation between various item scores and between item scores and total scale score of PHQ-9

	Item-1	Item-2	Item-3	Item-4	Item-5	Item-6	Item-7	Item-8	Item-9
Item-1	1.000	–	–	–	–	–	–	–	–
Item-2	0.660^**^	1.000	–	–	–	–	–	–	–
Item-3	0.434^**^	0.475^**^	1.000	–	–	–	–	–	–
Item-4	0.638^**^	0.531^**^	0.586^**^	1.000	–	–	–	–	–
Item-5	0.365^**^	0.354^**^	0.419^**^	0.450^**^	1.000	–	–	–	–
Item-6	0.566^**^	0.747^**^	0.423^**^	0.462^**^	0.387^**^	1.000	–	–	–
Item-7	0.608^**^	0.601^**^	0.426^**^	0.571^**^	0.382^**^	0.644^**^	1.000	–	–
Item-8	0.558^**^	0.556^**^	0.369^**^	0.503^**^	0.245^*^	0.549^**^	0.599^**^	1.000	–
Item-9	0.387^**^	0.527^**^	0.233^*^	0.268^**^	0.299^**^	0.513^**^	0.426^**^	0.438^**^	1.000
Total scores	0.789^**^	0.787^**^	0.647^**^	0.743^**^	0.567^**^	0.788^**^	0.769^**^	0.684^**^	0.569^**^

*PHQ-9* Patient Health Questionnaire

**P < 0.01 *P < 0.05

## Discussion

PHQ-9, a universal community screening tool for depression, is more likely to be used to measure the severity of depression in psychiatric hospitals. Indeed, the DSM-5 also recommends use of PHQ-9 as a tool for evaluating the severity of depression. All subjects in this study were clearly diagnosed as MDD using SCID (Structured clinical interview for DSM) to ensure the accuracy of diagnosis; the diagnosis was made during a disease episode or during remission.

Studies conducted in China as well as overseas have consistently shown that PHQ-9 has an I-factor structure, i.e., affective factor; in other words, all items in PHQ-9 measure the same concept [20, 26, 33]. Many other studies have also shown that PHQ-9 has II-factor structure: cognitive-affective factor and somatic factor. In this study, there was a strong correlation between HAMD-17 total scores and PHQ-9 total scores, which was consistent with previous findings [22–26]. These findings support the validity and feasibility of use of PHQ-9 for assessing depression severity.

In this study, we used HAMD scale scores of 7, 15, and 21 as cut-offs to designate mild, moderate, and severe symptom levels, respectively. This is slightly different from the cut-off scores used by the original developers of the scale. They recommended cut-off scores of 5, 10, 15, and 20 to designate mild, moderate, moderately severe, and severe depression, which is also more easily remembered by clinicians. There is no significant change in the reliability and validity of PHQ-9 to identify different severity levels of depression when the cut-off points changed within a small range.

HAMD-17 total scores and PHQ-9 total scores have good consistency, and there is general correlation between the disease severity as assessed by the two scales. This suggests that PHQ-9 can be used for rapid assessment of the severity of depression and for therapeutic monitoring. However, patients with severe depression require further assessment using HAMD.

Our findings of high internal consistency and high test-retest coefficient after 2 weeks are consistent with those of previous studies [23–28]. The correlation coefficient between the total score and each item score of PHQ-9 ranged from 0.572 to 0.813 (*P* < 0.01), which is indicative of strong correlation. Item 2 (feeling down, hopeless, or depressed) showed the strongest correlation with total score followed by item 1 (little pleasure or interest in doing things) and item 6 (feeling that you are a failure or bad about yourself or have let your family or yourself down). This suggests that these three items are most important determinants of the severity of disease. In this study, the PHQ-9 score showed the strongest correlation with mental factors and a relatively low correlation with somatic indicators. These results suggested that the PHQ-9 has enough discriminant validity for evaluating depression.

Limitations of the present study include the relatively small sample size. Further studies with a larger sample size may provide more definitive evidence. PHQ-9 assesses the changes in depression severity and is sensitive to changes in symptoms. However, according to a domestic research, PHQ-9 can be used to evaluate the psychological characteristics of patients with depression, but it is not sensitive to changes in symptoms [26]. We did not evaluate this aspect in the present study; this needs to be verified by incorporating appropriate study design in future.

## Conclusion

PHQ-9 showed good reliability and validity, and higher adaptability for patients with MDD in a psychiatric hospital sample. It is a simple, rapid, effective, and reliable measurement tool to screen depression and evaluate the severity of depression.

## Data Availability

Data are available from the first and the corresponding authors.

## Abbreviations

CI: Confidence interval
DALYs: Disability-adjusted life years
DSM-IV: Structured Clinical Interview for DSM Disorders-Fourth Edition
HAMD: Hamilton Depression Scale
ICC: Intraclass correlation coefficient
MDD: Major depressive disorder
PHQ-9: Patient Health Questionnaire-9
SCID: Structured Clinical Interview for DSM
